# Sequential analysis of surfactant, lung function and inflammation in cystic fibrosis patients

**DOI:** 10.1186/1465-9921-6-133

**Published:** 2005-11-07

**Authors:** Matthias Griese, Robert Essl, Reinhold Schmidt, Manfred Ballmann, Karl Paul, Ernst Rietschel, Felix Ratjen, the Beat Study Group

**Affiliations:** 1 Children's Hospital, University of Munich, Lindwurmstr 4, 80337 München, Germany; 2 Internal Medicine, University of Giessen, Klinikstr. 36, 35392 Giessen, Germany; 3 Department of Pediatric Pulmonology, Medical School, Carl-Neuberg-Str.1, 30625 Hannover, Germany; 4 Department of Pediatric Pulmonology and Immunology, Charité, Humboldt-University, Zum Heckeshorn 33, 14109 Berlin, Germany; 5 Department of Pediatric Pulmonology and Allergology, Children's Hospital, Josef Stelzmannstr.9, 50924 Köln, Germany; 6 Children's Hospital, University of Essen, Hufelandstrasse 55, 45122 Essen, Germany; 7 Principal investigators of the BEAT study group

**Keywords:** Surfactant protein, phospholipids, surface activity, pulsating bubble, capillary surfactometer, bronchoalveolar lavage, airway inflammation, lung function

## Abstract

**Background:**

In a cross-sectional analysis of cystic fibrosis (CF) patients with mild lung disease, reduced surfactant activity was correlated to increased neutrophilic airway inflammation, but not to lung function. So far, longitudinal measurements of surfactant function in CF patients are lacking and it remains unclear how these alterations relate to the progression of airway inflammation as well as decline in pulmonary function over time.

**Methods:**

As part of the BEAT trial, a longitudinal study to assess the course of airway inflammation in CF, we studied lung function, surfactant function and endobronchial inflammation using bronchoalveolar lavage fluid from 20 CF patients with normal pulmonary function (median FEV_1 _94% of predicted) at three times over a three year period.

**Results:**

There was a progressive loss of surfactant function, assessed as minimal surface tension. The decline in surfactant function was negatively correlated to an increase in neutrophilic inflammation and a decrease in lung function, assessed by FEV_1_, MEF_75/25%VC_, and MEF_25%VC_. The concentrations of the surfactant specific proteins A, C and D did not change, whereas SP-B increased during this time period.

**Conclusion:**

Our findings suggest a link between loss of surfactant function driven by progressive airway inflammation and loss of small airway function in CF patients with limited lung disease.

## Background

Cystic fibrosis (CF) is caused by mutations in the cystic fibrosis transmembrane regulator (CFTR) gene and early alterations are primarily found in the small airways [1]. Pulmonary surfactant is of critical importance for the maintenance of airspace patency during the respiratory cycle, especially at lower airway pressures at end-expiration. These biophysical functions are predominantly related to surfactant lipids [2,3], surfactant proteins (SP-) B and -C, as well as SP-A which contributes to resistance against inhibition of surfactant activity by products of inflammation. Altered surfactant has been associated with impaired lung function in young children with respiratory infections [4,5]. Thus, derangements of any of these components may affect the biophysical surfactant activity and may contribute to early alterations of lung function in patients with cystic fibrosis [6].

In a previous cross-sectional analysis of patients from the BEAT (Broncho-alveolar Lavage for the Evaluation of Anti-inflammatory Treatment) study, a long term study using bronchoalveolar lavage (BAL) to assess the evolution of airway inflammation in CF patients with normal lung function, we have observed a reduction of surfactant activity reflecting impaired airway patency in the majority of these CF patients [7]. The degree of surfactant dysfunction was inversely correlated with neutrophilic endobronchial inflammation, but no correlation was observed with flows at low lung volumes (MEF25_%VC _or MEF_25/75%VC_).

Flows at low lung volumes have been used to assess these early functional abnormalities, but due to inhomogeneous distribution of disease manifestation and a high inter-individual variability, they have not been found to be particularly sensitive when used cross-sectionally [8,9]. Longitudinal assessment of lung function is more sensitive in detecting deviations from personal best values [10,11]. Thus, sequential analyses appear to be more powerful to detect pathological changes [12,13]. We hypothesized that if surfactant function is relevant for small airway disease in CF patients, functional abnormalities in surfactant should be related to deterioration in pulmonary function assessed longitudinally. Some results of these studies have been reported previously in the form of an abstract [14].

## Materials and methods

### Patients and samples

Patients with cystic fibrosis were recruited from the BEAT study, a multi-center study using bronchoalveolar lavage to evaluate the evolution of airway inflammation in CF and its modulation by rhDNase treatment [15]. Inclusion criteria were an established diagnosis of CF, the ability to perform lung function tests, normal lung function defined as a FEV_1 _> 80% predicted, and absence of acute respiratory tract infections prior to bronchoscopy for at least 6 weeks. Patients receiving anti-inflammatory treatment (ibuprofen and systemic or inhaled corticosteroids), and those with allergic bronchopulmonary aspergillosis were excluded. The local ethic committees of all participating centers approved the study; written informed consent by both parents and/or patients was obtained in all cases. BAL was performed prior to randomization, after 1.5 as well as after 3 years in the same segment/subsegment over time. For this study samples from all patients were considered in whom 3 BALs were performed and sufficient material was available for analysis. More than 5 ml of BAL fluid at all 3 time points was available in 37 of 105 subjects. The measurement of surfactant proteins and functional assessment in the capillary surfactometer required 2.5 ml, therefore 2.5 ml could be used for surface tensions measurements in the bubble surfactometer. Since this technique requires at least 40 μg of phospholipids, phospholipid concentration had to be 16 μg/ml, which was the case in 22 subjects. Two samples of two different subjects were lost during processing; therefore 20 patients (13 females) with a median age of 10 years (range 5–16) were left for the final analysis. 13 of the patients were dF508 homozygeous, 3 were dF508 compound heterozygeous, in 2 patients other mutations were present, and in 2 the mutations were unknown. Four of the 20 patients were positive for P. aeruginosa. FVC was 91% pred. (70–126), FEV_1 _94% pred. (76–118), MEF_25%VC _69% pred. (27–155)). The status of infection, i.e. the type of bacteria recovered from the airway specimens and serum values for C-reactive protein, total IgG and neutrophils were the same at all 3 time points assessed. The subpopulation did not differ from the total study population in both pulmonary function and baseline BAL parameters. 9 of 20 patients were randomized to rhDNase once daily. Because of the small number of patients in these subgroups, a sub-analysis related to rhDNase treatment was not performed in this study. For comparison to the levels in normal children, the mean and range of the biophysical and cellular measures in healthy children are indicated in the figures where appropriate. These and additional data obtained with the same methods can be found in a previous publication [7].

### Bronchoalveolar lavage (BAL), sample preparation and biochemical and biophysical measurements

BAL was performed as described with 3 × 1 ml/kg body weight normal saline warmed to body temperature [15,16]. The first aliquot of the recovered BAL fluid was treated separately; all other samples were pooled for analysis. The total cell count was measured in a hemocytometer, the differential cell count was assessed from cytoprep slides. Aliquots of the cell free BAL supernatant of the pooled BAL sample were used for the analysis of total protein, and the surfactant proteins by ELISA (SP-A, SP-D [17] and SP-B, SP-C [18,19]). Pulmonary surfactant may be differentiated into a very surface active fraction, i.e. the large surfactant aggregates (LA) and a less active fraction containing smaller surfactant particles (small aggregates, SA), which may also inhibit surfactant function. 5 ml aliquots of the BAL fluid were centrifuged at 40,000 × g for 30 min to generate a surfactant pellet, containing the LA and the supernatant, containing the SA [19]. The phospholipid content was determined by phosphorus assay of a lipid extract of the surfactant pellet [19]. After resuspension of the surfactant pellet (NaCl 140 mM, HEPES 10 mM, EDTA 0.5 mM, CaCl_2 _3.5 mM, pH 6.9) the surface activity was assessed in a pulsating bubble surfactometer at a final phospholipid concentration of 1 mg/ml [19]. In addition, the biophysical activity was assessed in a capillary surfactometer [20]. Briefly, glass capillaries (0.255 mm internal diameter at its most narrow part) were filled with 0.5 μl of the above described surfactant suspension and assessed for the capability at 37°C to keep the capillary open over a time period of 120 seconds. Water (0% of the time open) and a purified bovine surfactant (96.7% of the time open) served as a control [20].

**Figure 1:**
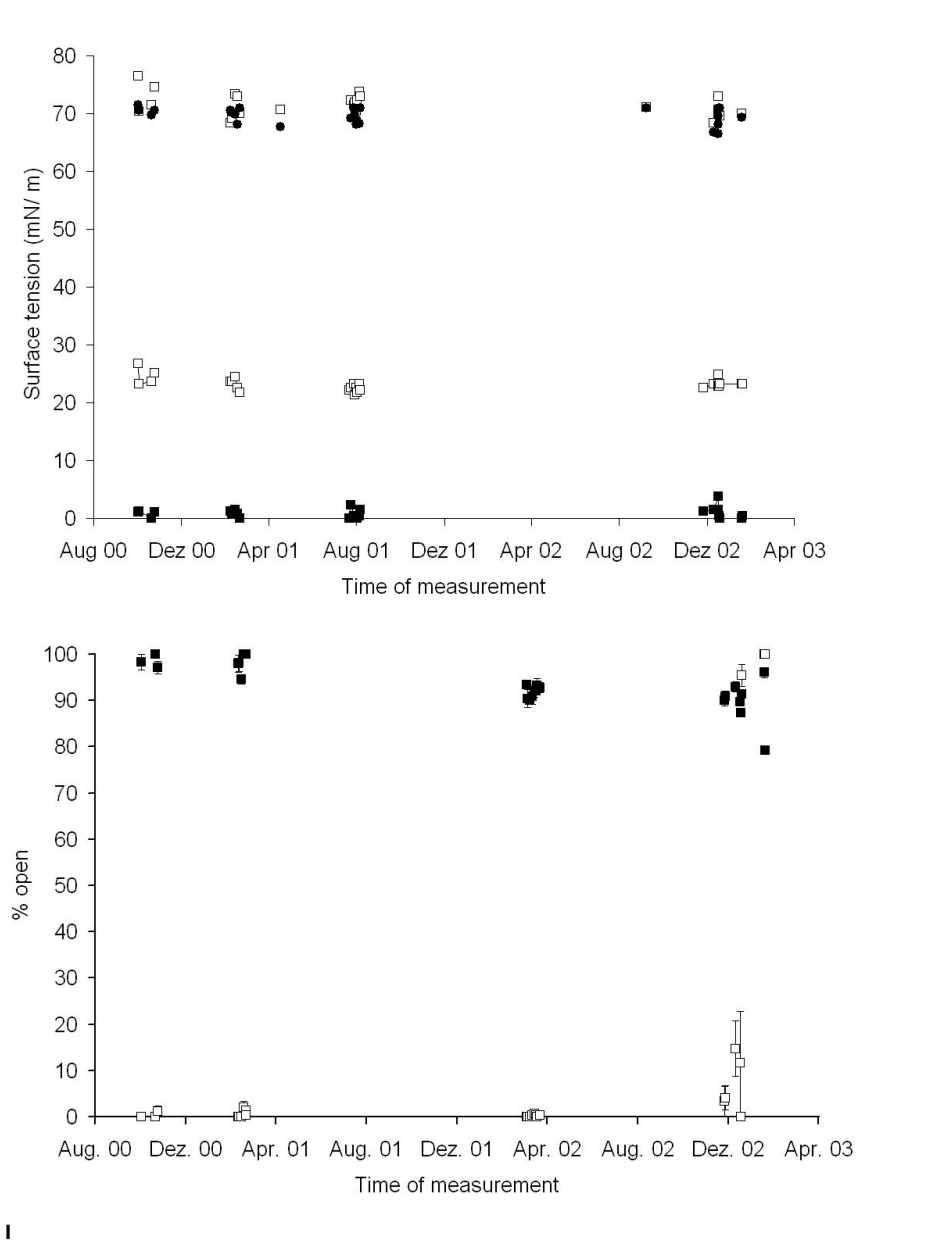
Quality control assurance by the assessment the function of a bovine surfactant (minimal surface tension, closed squares; surface tension after adsorption, open squares) and of water (minimal surface tension, closed circles; surface tension after adsorption, open squares) in the pulsating bubble surfactometer (upper panel) and the capillary surfactometer (lower panel) (bovine surfactant, closed squares, water, open squares) over a period of more than two years.

Reliability and precision of the measurements of surface activity was assured by the regular assessment of a well functioning bovine surfactant, water and methanol each working day (Fig. 1). Appropriate and stable readings were obtained over a period of more than 2 years for the pulsating bubble surfactometer and also the capillary surfactometer, except for two days with high readings in the capillary surfactometer towards the end of the study (Fig. 1, lower panel). These high readings were related to problems with the diameter of the capillaries and new batches, which gave the expected lower values, were used.

### Statistical analysis

The nonparametric ANOVA, i.e. Friedman test was used for comparison of repeated measured values over the study period at the 3 time points, followed by the Dunn's post-hoc test to detect differences between each time points. Correlations were calculated with the Spearman rank test. Non-paired comparisons were made with the nonparametric Mann-Whitney test. The results are given as medians and ranges or as individual values (mean of 2 to 4 determinations). A p level of <0.05 was considered statistically significant. Statistical analysis was done with Prism 4.

## Results

**Figure 2:**
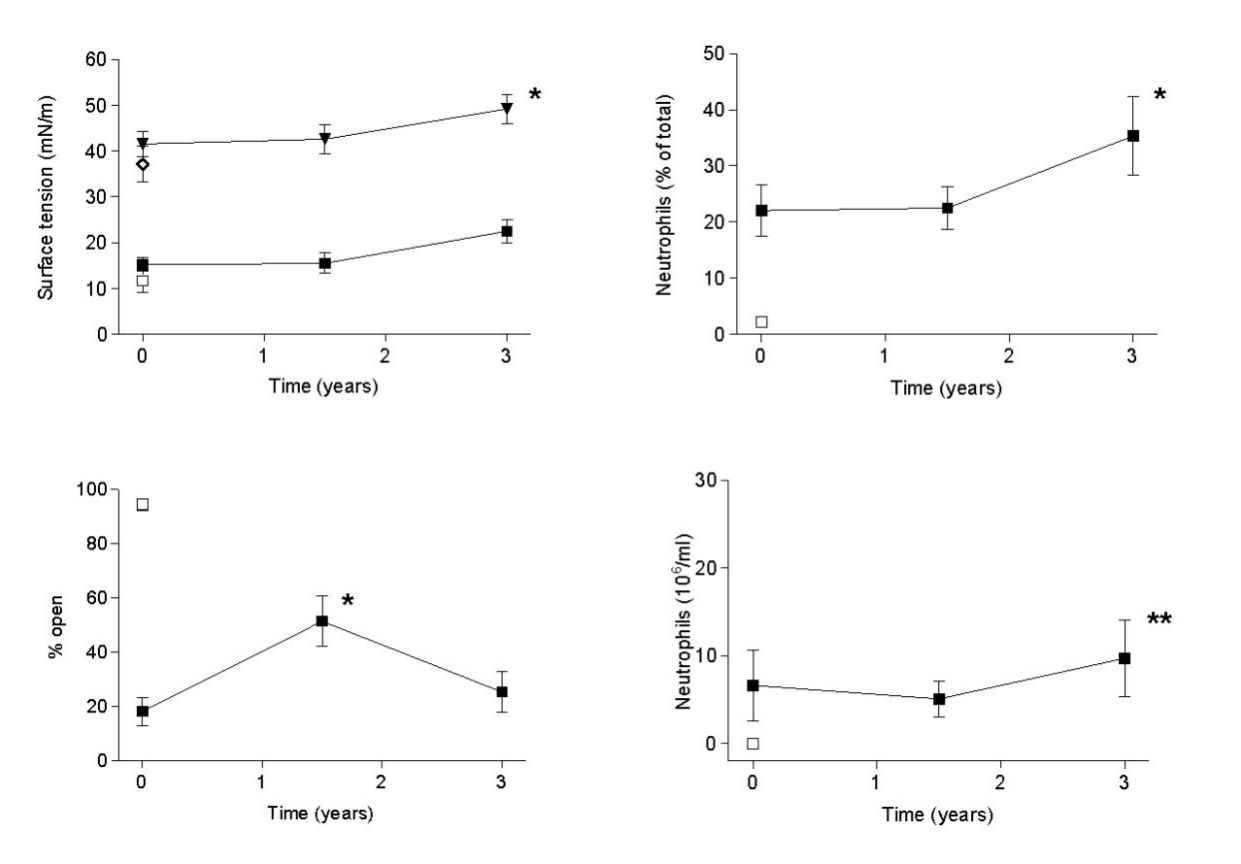
Surface activity of a surfactant fraction obtained from bronchoalveolar lavages in 20 patients with cystic fibrosis assessed in a pulsating bubble surfactometer (upper left panel), i.e. expressed as surface tension at minimum bubble radius (squares) and surface tension at adsorption (triangles), assessed in a capillary surfactometer (lower left panel), i.e. expressed as the percentage of time open of a small capillary of total time. The percentage of neutrophils (upper right panel) and the absolute number of neutrophils (lower right panel) as markers of inflammation in their BAL fluids are also indicated. Data are given as mean and standard error of the mean. Differences between the different time points were assessed by nonparametric Friedman ANOVA, followed by Dunn's post hoc multiple comparison test. The asterisks indicates a significantly different value, compared to the first data point (* P < 0.05, ** P < 0.01). The open symbols represent values in normal subjects assessed with the same methodolgy [7,29].

**Figure 3:**
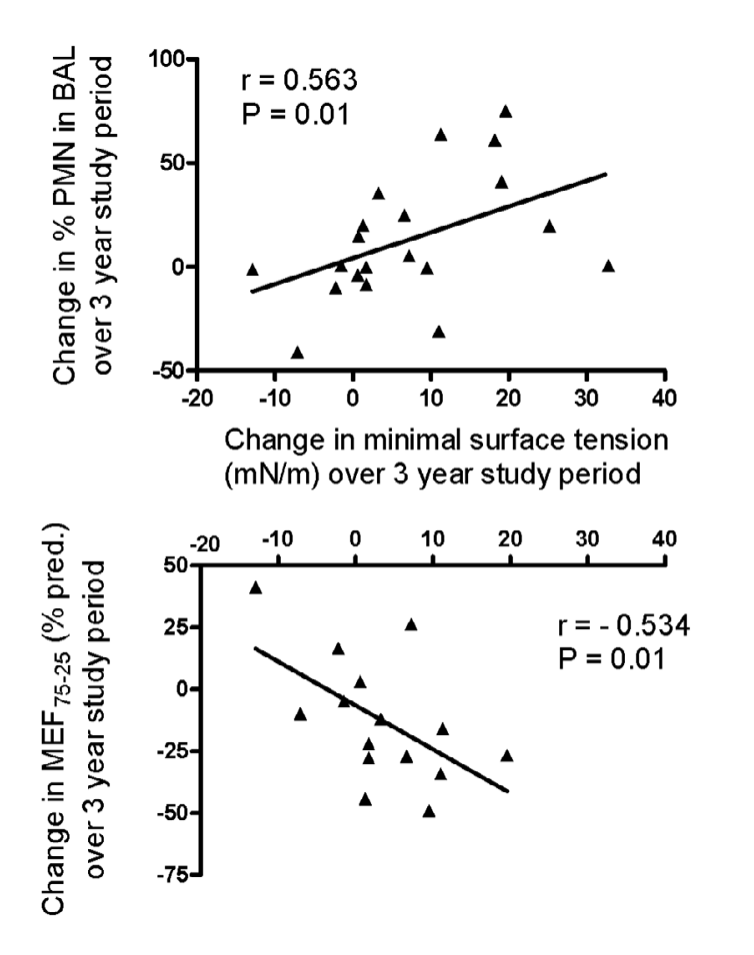
Correlation of the change in surfactant activity, assessed as minimal surface tension, to the change of the fraction of polymorphonuclear leukocytes in BAL (upper panel) and to the change of small airway function, assessed as MEF_75/25 _(lower panel). Changes were calculated as the difference between the last visit at 3 years and the first visit at start of the study. Spearman rank correlations and linear regression line are indicated.

Minimum surface tension increased, reflecting deteriorating function, over the 3-year period (Fig. 2, left upper panel). This was paralleled by an increase in the relative and total number of neutrophils (Fig. 2, right panels). The increase of minimum surface tension over the 3 years was directly proportional to an increase in neutrophils (Fig. 3, upper panel). The total amount of phospholipids in BAL fluid was higher in the initial BAL than in the two following studies (Tab. 1). This finding is not responsible for the observed loss of surfactant function, which was assessed at a fixed phospholipid concentration. Surfactant function assessed in the capillary surfactometer, which was already severely reduced at baseline compared to values in healthy children, improved slightly after 1.5 years, but did not change significantly over the 3 year period (Fig. 2).

**Table 1:** Surfactant proteins (SP-) A, B, C and D, total proteins and phospholipids in three consecutive bronchoalveolar lavages (BAL) in patients with cystic fibrosis over a 3 year period

	1. BAL at start of the study	2. BAL after 1.5 years	3. BAL after 3 years
SP-A (ng/ml)	5312.0 (2409.0 – 13218.0)	5580.0 (1334.0 – 13048.0)	3782.0 (128.6 – 13624.0)
SP-B (ng/ml)	397.5 (247.2 – 1219.0)	897.5 (472.5 – 1252.0)†	847.0 (375.5 – 1602.0) **
SP-C (ng/ml)	635.0 (37.0 – 1557.0)	638.7 (391.5 – 2012.0)	581.2 (15.5 – 1489.0)
SP-D (ng/ml)	7.41 (1.17 – 22.06)	5.41 (1.48 – 43.46)	6.25 (0.00 – 48.21)
Protein (μg/ml)	104.70 (52.60 – 382.40)	97.88 (46.60 – 193.40)	94.20 (38.44 – 258.80)
Phospholipids (μg/ml)	50.37 (19.45 – 102.50)	33.56 (20.75 – 429.40)	32.64 (14.84 – 70.00) **

Comparisons were made by Friedman test followed by Dunn's post hoc test. Differences between the beginning and the end of the study are indicated by **, differences between the first and second BAL are indicated by †. Level of significance († P < 0.05, ** P < 0.01). Data are median (range) of 20 patients.

**Figure 4:**
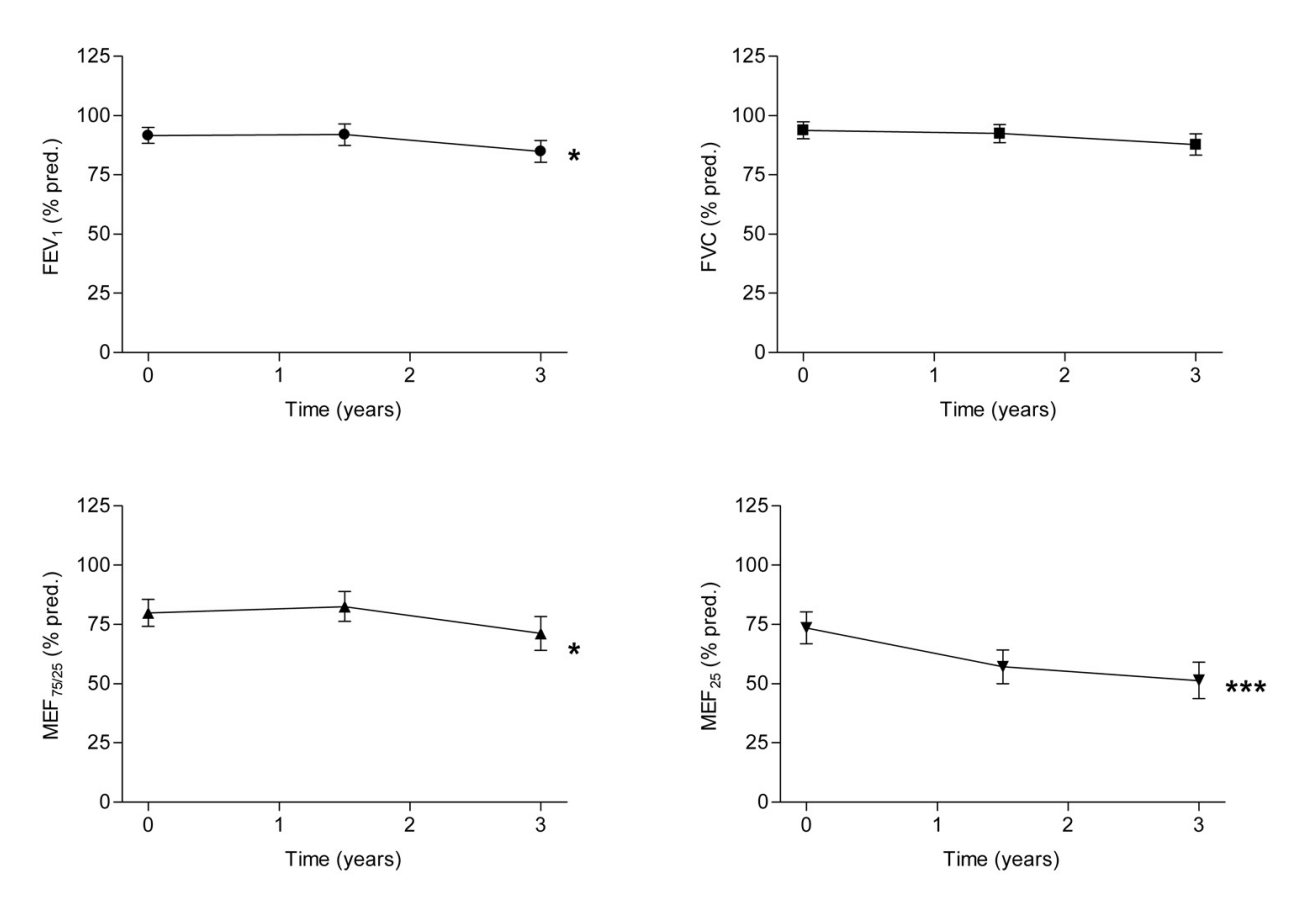
Lung function over time in 20 subjects with CF. FEV_1_, MEF_72/25%VC _and MEF_25%VC _were significantly lower 3 years after start of the study (Friedman ANOVA, followed by Dunn' post hoc multiple comparison test, * p < 0.05, *** p < 0.001). FVC did not change.

Lung function assessed as FEV_1 _significantly deteriorated over time (Fig. 4, upper panel). This trend was even more pronounced for lung function parameters representing predominantly the small airways (MEF_75/25 _and MEF_25_) (Fig. 4, lower panels). The changes of all three measures of airway obstruction used here, were negatively correlated to an increase of minimal surface tension (FEV_1 _r = -0.560, P = 0.009, MEF_25%VC _r = -0.536, P = 0.015 and MEF_75/25%VC _see Fig. 3, lower panel).

The concentration of the surfactant specific proteins SP-A, C and D, as well as the total protein content of BAL fluid did not change significantly, whereas SP-B was higher after 1.5 and 3 years than at the beginning (Table 1).

In the absence of reduced concentrations of the surfactant proteins, proteolytic attack resulting in inactivation and the presence of fragments of the proteins, may also be responsible for dysfunction of surfactant [21,22]. No such fragments were found by Western blotting for SP-B and SP-C in 21 CF patients over the range of neutrophilic inflammation, present in this cohort (Tafel, Latzin and Griese, unpublished observations). BALs from 15 CF patients from this cohort were screened for proteolytic fragments of SP-A. In 3 subjects weak bands were found; one band at 23 kD, one at 18 kD and in another subject one at 16 kD. These bands contributed to less than 1% of the total SP-A present on each of the blots (Wassilewa and Griese, unpublished data).

**Figure 5:**
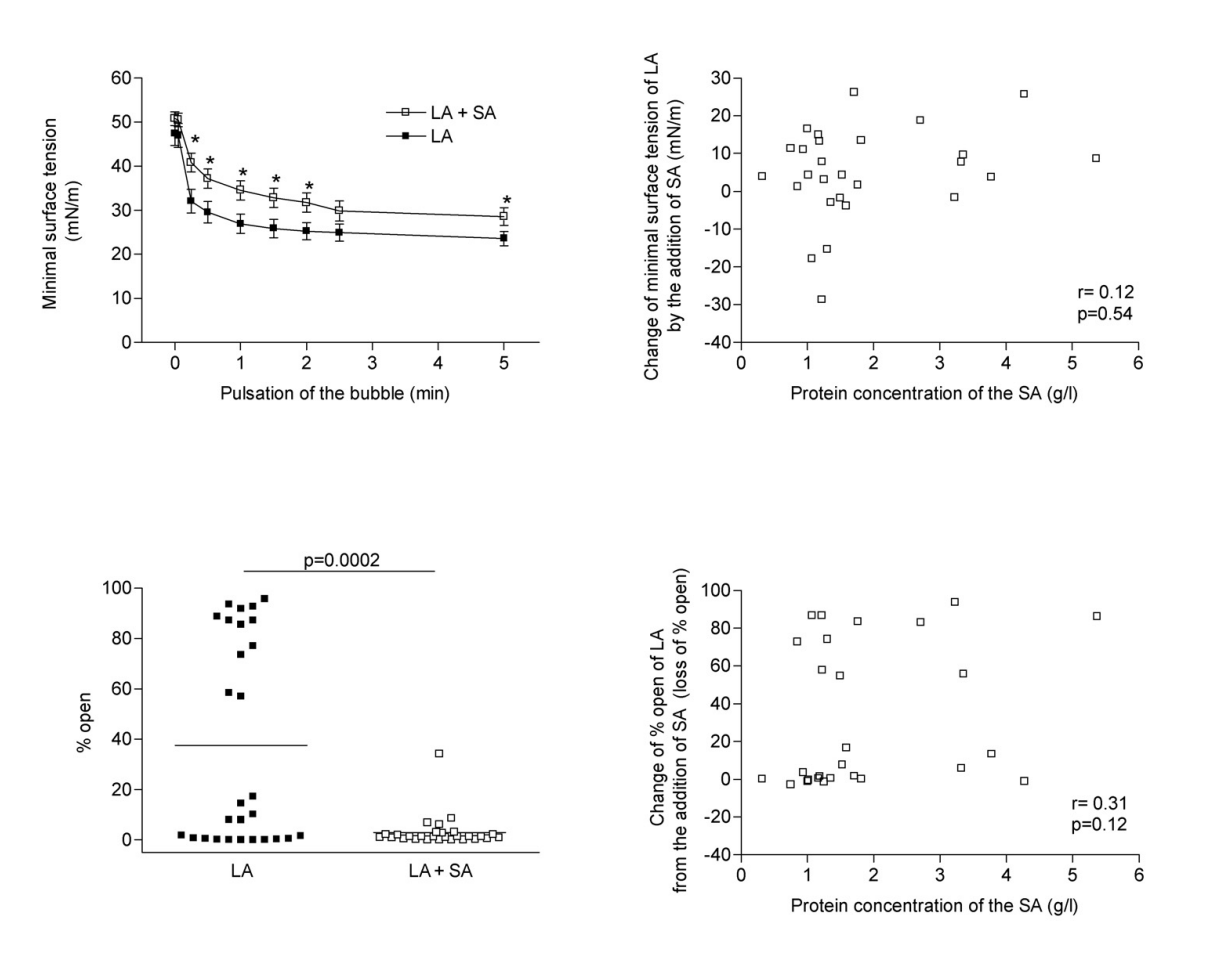
Surfactant activity assessed in the pulsating bubble surfactometer (upper panels) and the capillary surfactometer (lower panels). The large aggregate (LA) surfactant fraction was recombined with the appropriate small aggregates (SA) of surfactant, demonstrating significant inhibitory activity (left side panels). This inhibitory activity of SA was not dependent on its amount, i.e. was not the sole determinate of surface activity (right side panels). Comparisons by Mann-Whitney test and correlations by Spearman rank test.

The surface active fraction of pulmonary surfactant, i.e. the large surfactant aggregates (LA) can be separated from the less active small aggregates, i.e. SA, which may also inhibit surfactant function. We next investigated the contribution of the SA fraction to overall surfactant function. SA added to LA at the original ratio present in bronchoalveolar lavage, significantly reduced surfactant function both, in the pulsating and in the capillary surfactometer (Fig. 5, left panels). Interestingly, this effect was not related to the protein content of SA (Fig. 5, right panels). To clarify this issue further, we asked the question, if the SA protein which has a similar protein composition as serum [21], differed from serum in its potency to inhibit surfactant, i.e. from the presence of components that have an intrinsically higher capacity to inhibit surfactant function. No such differences were found at two representative concentrations of SA investigated (Tab. 2).

**Table 2:** Effect of small aggregates (SA) or serum on the surface activity of a well functioning proving surfactant, assessed in a pulsating bubble surfactometer and in a capillary surfactometer

	Pulsating bubble surfactometer	P	Capillary surfactometer	P
	Minimal surface tension (mN/m)		% open	
Bovine surfactant	2.3 (0.0 – 3.50) 5		100.0 (99.9 – 100.0) 5	
+ 1.5 g/l SA protein	2.3 (1.5 – 6.6) 5	ns	3.35 (0.2 – 10.3) 5	ns
+ 1.5 g/l serum protein	1.9 (1.1 – 8.6) 6		0.95 (0.1 – 8.5) 6	
+ 4.5 g/l SA protein	8.6 (2.3 – 24.6) 5	ns	1.03 (0.6 – 2.3) 5	ns
+ 4.5 g/l serum protein	19.6 (1.5 – 31.9) 7		4.83 (0.3 – 35.5) 7	

Data are medians (range) from n determination. Comparisons between the effects of the addition of serum and SA protein were done by Wilcoxon test.

## Discussion

This is the first study to assess surfactant function, lung function and airway inflammation longitudinally in CF patients. The results demonstrate loss of surfactant function over time parallel to an increase in neutrophilic inflammation and a decrease in small airway function. These data support the hypothesis that inflammation leads to disturbances of the local airspace environment, including properties of the surfactant system resulting in a loss of patency of small airways.

The mechanism(s) responsible for this loss of surfactant function remain to be resolved. An altered phospholipid composition could contribute to reduced surface activity, but this appears rather unlikely, as no significant deviations have previously been found in a group of younger CF patients [4,23]. Similarly, no age dependency of the four surfactant proteins, total phospholipid and total protein, was observed in the whole cohort of CF patients from the BEAT study spanning an age range from 5 to 31 years (n = 75) [7]. Here we did not find reductions in the concentrations of the four surfactant proteins over time, suggesting that the loss of surface activity cannot be easily explained by a single factor like an altered expression of one of the surfactant proteins. Alterations of the integrity and function of the surfactant proteins without a change in their concentration, such as by proteolytic degradation, were largely excluded as we found no such fragments for SP-B and SP-C (Tafel, Latzin and Griese, unpublished data). For SP-A in 3 out of 15 investigated patients very small amounts of SP-A degradation products (<1% of total SP-A present) were detected by Western blotting, which was unlikely to be of functional relevance (Wassilewa and Griese, unpublished data). In addition to proteolytic damage, oxidative modification of the proteins may occur and contribute to surfactant protein dysfunction, however this was not assessed systematically in this cohort [21,22,24,25]. Little pronounced, but continuously ongoing inflammatory processes may gradually evoke changes in the function of the surface active fraction of surfactant, which may be responsible for the observed loss of biophysical surfactant activity. The principal surface active fraction of pulmonary surfactant recovered from the airspaces is represented the large surfactant aggregates (LA). Early changes in the LA surfactant micro-texture like an increased admixture of inactivating components from the SA fraction with enhanced local inflammation could be involved [26]. This explanation does not necessitate changes in the intrinsic inhibitory potency of the SA proteins and is in accordance with our results that SA fractions did not differ in their surfactant function and had a similar inhibitory potency as serum.

In our previous cross-sectional study of the same cohort of CF patients, we observed only a marginally decreased surfactant function assessed in the pulsating bubble surfactometer [7]. When those samples were analysed in the more sensitive capillary surfactometer, which simulates a small airway, the functional capacity of the surfactant was more extensively impaired with a mean capillary openness below 20% [7]. Open small airways are critical for the reduction of overinflation, the deposition of anti-inflammatory and anti-microbial treatments in those areas of the lungs where the initial neutrophilic inflammation begins to destroy the lung's integrity of patients with CF. The lack of change over time observed in this study with the capillary surfactometer may be caused by this marked reduction observed already at baseline rendering this methodology too sensitive to detect further impairments in surfactant function over time.

Of interest is a lack of correlation between minimal surface tension assessed in the pulsating bubble surfactometer and the % open assessed in the capillary surfacometer (r = -0.060; P = 0.768), when the same samples were assessed. Also, the changes of these variables over time did not correlate with each other. Identical results were obtained previously in the larger BEAT cohort (n = 75) investigated similarly for these two variables (r = -0.140; P = 0.095) [7]. Together these data demonstrate that the two techniques have different sensitivities and may also measure two distinct aspects of surfactant activity of airway specimens.

Using the pulsating bubble surfactometer and assessing minimal surface tension, loss of surfactant activity was detected with time. The concordance of the increase of minimal surface tension, loss of lung function, including small airways function and their inverse correlation support the concept that the surfactant function may be relevant for the stability of small airways [20,27]. Thus treatment strategies directed to keep the small airways patent, i.e. physiotherapeutic interventions maintaining a positive expiratory pressure or inhalation of exogenous surfactant [28] may be relevant for the treatment of early CF lung disease.

There are some important limitations of this study that need to be taken into account when interpreting the data. First, the number of patients reported in this cohort is relatively small and consists of a subgroup of the total study cohort [16]. However, we found no significant differences in CFTR genotype, sex, baseline lung function, bacterial colonisation and BAL neutrophils at baseline between the cohort reported here and the total BEAT study population. In the original report of the BEAT study results, we have observed an inhibiting effect of rhDNase on airway inflammation over time and this may also influence the data reported here. When we compared the changes in a subgroup analysis, we did not observe any differences in change of surfactant function over time between the 2 groups, but this finding has to be interpreted with caution, because the number of patients included is too small to detect effects of rhDNase. Similarly, the other variables assessed here, i.e. surfactant function and airspace inflammation were not different between the subgroups. Finally, bacterial infection changes over time and this may impact upon both inflammation and surfactant function. Again, subgroup analysis is problematic because of small numbers. The change in surfactant function showed no relationship to changes in bacterial colonisation, which may be related to the fact that most patients were already infected with CF pathogens. The effect of bacterial infection should therefore ideally be studied in a population which does not have airway infection initially.

Our data are in agreement with previous cross-sectional studies in children with CF including patients with impaired lung function, where minimal surface tension was reduced, whereas the concentrations of total phospholipids and of dipalmitoyl phosphatidylcholine were within the normal range [4,23]. Similar observations were made in older children and young adults [19], suggesting that there may be an early, but secondary, derangement of the biophysical surfactant properties in CF. With this longitudinal study of CF patients we hoped to eliminate a substantial fraction of the variability by investigating the changes within individuals overtime. Despite a large cohort of patients to start with [16], eventually only 20 complete triplets of sufficient BAL fluid were available for all the biophysical measurements. Nevertheless, we found a clear increase in minimal surface tension over the time period investigated and also correlations between these changes, the changes in lung function and an increase in neutrophils.

In summary, pulmonary surfactant function, lung function and airway inflammation were assessed sequentially over a 3-year period in CF patients with initial lung function within the normal range. An increase in neutrophil inflammation over time was associated with a decrease in small airway function and a loss of surfactant activity. These findings highlight the importance of early changes in small airway biology and strongly suggest that specific therapeutic interventions targeting this region of the lung are warranted.

## Competing interests

The author(s) declare that they have no competing interests.

## Authors' contributions

MG designed the experiments on the BAL samples collected in the clinical study, drafted the manuscript. RE carried out the biophysical and the biochemical measurements, did the data calculations, performed the statistical analysis and participated in the drafting of the manuscript. RS carried out the SP-B and SP-C immunoassays. FR together with MG, MB, ER and KP conceived the study, participated in its design, coordination, practical realization and helped to draft the manuscript. All authors read and approved the final manuscript.
